# Feasibility of Generatively Designed Patient-Specific Implants for Orthognathic Surgery: An In Silico Comparative Study

**DOI:** 10.1016/j.identj.2026.111095

**Published:** 2026-09-02

**Authors:** Mengjia Cheng, Ankit Nayak, Yiu Yan Leung

**Affiliations:** a Oral and Maxillofacial Surgery, Faculty of Dentistry, The University of Hong Kong, Hong Kong SAR, China; b Department of Mechanical Engineering, School of Advanced Engineering, UPES, Energy Acres, Dehradun, India

**Keywords:** Orthognathic surgery, Computer-aided design, Computer-aided manufacturing, Skeletal fixation, Patient-specific implants, Generative design

## Abstract

**Introduction and Aims:**

To investigate whether generatively designed (GD) patient-specific implants are equivalent to manually designed (MD) PSIs for orthognathic surgery in the in silico setting.

**Methods:**

Twelve pairs of GD and MD fixation plates for Le Fort I osteotomy and genioplasty were compared regarding plate-bone shape fit, biomechanical performance, plate size, and design time. Shape fit was measured as the signed distance from the plate vertices to the underlying bone surface to estimate potential surgical accuracy. Biomechanical performance (von Mises stress and deformation) was analysed using finite element analysis. Plate size was quantified by both surface area and volume. Hands-on design time was recorded for GD by stage, and historical data on MD time were referenced for context.

**Results:**

GD plates demonstrated better shape fit, with a lower mean plate-bone distance (0.95 ± 0.17 mm) than MD plates (1.12 ± 0.28 mm; *P* < .05). The maximum von Mises stresses of both plates were within the ultimate tensile strength of titanium (345 MPa), and the greatest deformations were within 0.55 mm, demonstrating sufficient biomechanical stability. GD plates reduced the surface area by 16% (*P* < .001) and volume by 18% (*P* < .05) compared to MD plates. Average hands-on design time for GD plates was 94.3 ± 14.2 minutes for Le Fort I and 45.0 ± 7.3 minutes for genioplasty, which was substantially shorter than MD time reported in prior in-house workflows.

**Conclusions:**

GD fixation plates demonstrated improved shape fit, adequate mechanical strength, smaller size, and reduced design time compared with MD plates.

**Clinical Relevance:**

The objective analysis of GD fixation plates in silico is important for justifying further application in clinical trials.

## Introduction

In recent years, the application of patient-specific implants (PSIs) has significantly enhanced the execution and accuracy of orthognathic surgery (OGS).^1^^,^^2^ PSIs, including surgical guides and bone fixation plates, are personalised based on individual facial skeleton features and preoperative surgical plans. The PSI design process involves extensive manual drawing, requiring high-level computer-aided design (CAD) expertise and long design duration. Typically, they are manually designed (MD) by specialised engineers in commercial companies or hospitals using professional CAD software. Consequently, the use of PSIs in OGS often results in significantly increased costs and extended turnaround times for patients and hospitals compared with the wafer approach.^3^^,^^4^ Moreover, effective communication between surgeons and engineers is essential to ensure PSI designs meet surgical procedural factors and surgeon preferences. These drawbacks have hindered PSIs’ widespread adoption in OGS.^3^

To reduce cost and turnaround time, Leung et al^5^ proposed in-house surgeon self-designed PSIs for OGS. The study demonstrated the feasibility and convenience of a surgeon-led, in-house PSI design modality. However, mastering CAD software is still technically challenging and time-consuming for surgeons. To make the process more accessible, our group recently developed a semiautomatic PSI design workflow based on generative design (GD) technology. This workflow leverages both geometric and biomechanical parameters to automatically generate and optimise fixation plates, offering a more efficient and user-friendly alternative to traditional manual design.^6^ Initial tests on single cases of Le Fort I osteotomy and genioplasty showed that the GD workflow produced fixation plates more compact than their MD counterparts while still presenting adequate mechanical strength. However, the properties of the GD fixation plates must be thoroughly evaluated to assess their clinical feasibility. Additionally, the generalisability of the GD workflow needs to be further validated using a broader range of OGS cases.

Hence, we conducted this in silico comparative study to assess GD fixation plates against MD fixation plates across a series of diverse clinical OGS cases. Given the dual role of fixation plates in OGS—guiding bone repositioning and providing stable fixation, plate-bone shape fit and biomechanical performance would be the primary outcomes. We hypothesised that GD fixation plates would be equivalent to MD plates in these key metrics in the in silico setting. Additionally, plate size and design time would be evaluated to further explore the practical advantages of the GD workflow.

## Materials and methods

**Fig. 1:**
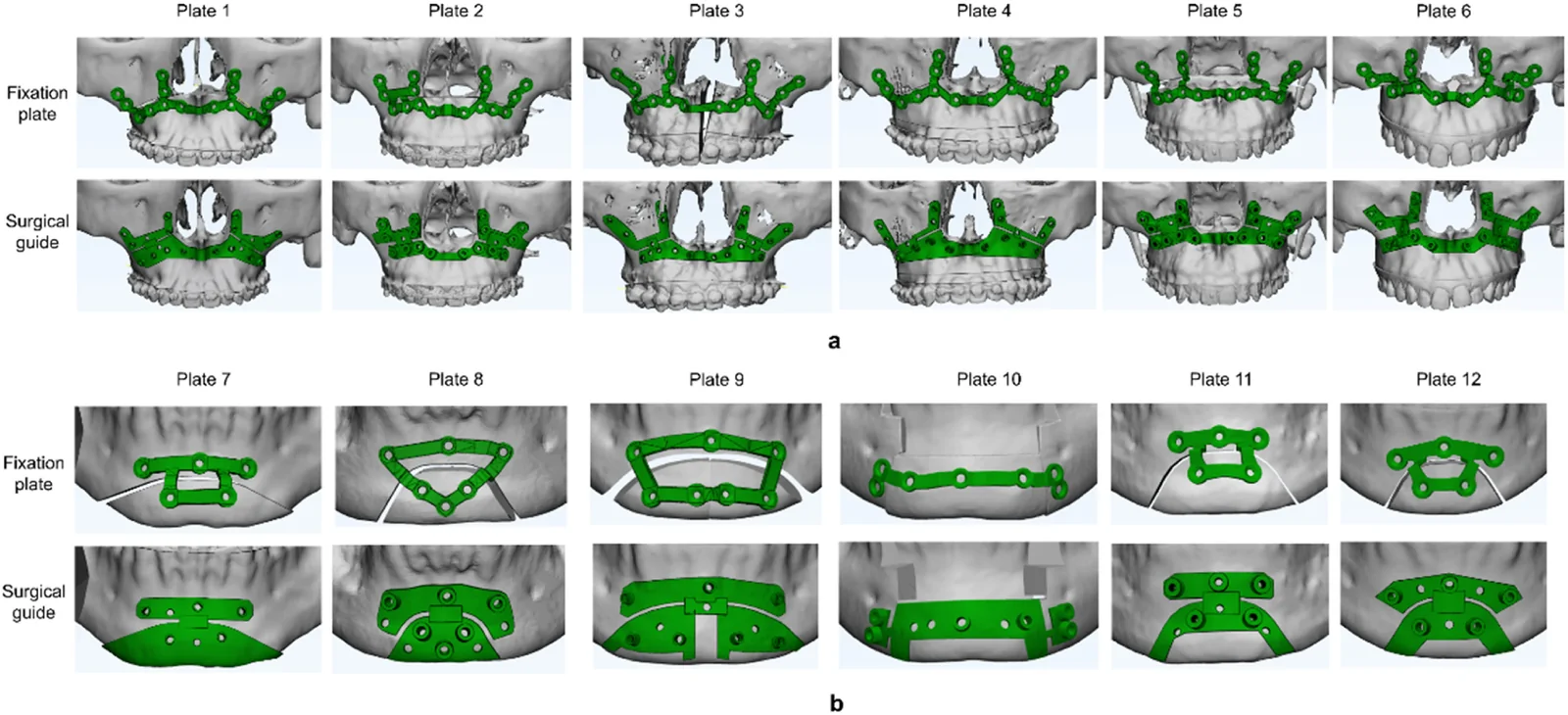
Manually designed patient-specific implants (PSIs) and underlying bone shape of selected cases. a, Le Fort I PSIs. b, Genioplasty PSIs.

Twelve OGS cases were retrospectively selected from the Division of Oral and Maxillofacial Surgery, Faculty of Dentistry, the University of Hong Kong, Hong Kong. All patients had undergone OGS using in-house surgeon-designed PSIs as the protocol in Leung et al.^5^ No formal power and sample size calculation was performed due to the exploratory nature and limited case availability. However, a sample size of 12 per group was considered sufficient for this feasibility assessment as it aligns with established statistical recommendations for pilot studies.^7^^,^^8^ Furthermore, this sample size allowed us to adequately stress-test the GD workflow by purposively selecting cases to maximise diversity in plate geometry and osteotomies, irrespective of their clinical application frequency (Figure 1). Six of them had Le Fort I osteotomy, and 6 had genioplasty. This selection was intended to assess the generalisability of the GD-based workflow. Retrieved data included digital files of virtual surgical planning (VSP) and the MD PSIs.

### PSIs design methods

#### MD PSI design protocol

MD PSIs were designed by oral and maxillofacial surgery trainees in our centre who were proficient in the workflow described previously.^9^ The workflow is summarised as below. The VSP was performed in Proplan CMF software (Materialise). Screws, represented by 16-mm-long cylinders (1 mm diameter) were inserted and adjusted to appropriate positions in Proplan CMF, ensuring no interference with any vital anatomic structures and being supported by adequate bone. Then, the simulated postoperative jaw segments and screws were imported into a CAD software (3-Matic; Materialise). The rings and connecting bars were manually modelled as the components of fixation plates. All components were finely adjusted to fit the bone shape and then merged to complete a fixation plate. Surgical guides were designed by subtracting osteotomy planes and adding drill holes on a wrapped planar shape.

#### GD PSI design protocol

GD PSIs were designed in Fusion (Autodesk) by M.C. following a detailed protocol previously published as a technical note.^6^ To provide a brief overview of the workflow, the design process began with the manual positioning of ring components as the screw holes on the underlying bone. To make the results comparable, the screws and rings in the template were set to the same specifications as the manual approach. Within the GD workspace, the rings were defined as preserve geometries while the underlying bone shape and screws were defined as obstacle geometries. Structural loads and constraints were then applied. The design objective was set to “minimise mass,” and minimum thickness was set as 1.0 mm. “Additive manufacturing” was chosen as the manufacturing method, with material specified as pure titanium. The programme then automatically connected the rings to generate multiple plate options. To meet surgeons’ preference for complex shapes in some cases (plates 8 and 9), the starting shape generated by “automated modelling” was leveraged in GD computation to ensure comparable results with MD plates. Surgical guides were designed in Fusion as well, but GD computation was not involved in their design.

### Outcome evaluation

The primary outcomes included plate-bone shape fit and biomechanical performance (von Mises stress and deformation), selected for their direct relevance to the clinical performance and safety of fixation plates. Shape fit determines how well the plate conforms to the bone surface, which can influence the accuracy of bone segment positioning. Maximum von Mises stress indicates the risk of material failure under functional loading, while maximum deformation reflects the likelihood of undesired movement or instability at the osteotomy site. Secondary outcomes were plate size and design time, selected for potential cost-effectiveness evaluation. Plate size, including surface area and volume, was calculated using the CloudCompare software. Due to the retrospective nature of data collection, design time was recorded exclusively for GD PSIs. However, the MD times based on self-reported estimates at our centre would be referenced for context. The following sections described data preprocessing steps and the detailed methodologies for evaluating shape fit and biomechanical performance.

#### Data preprocessing

MD and GD plates were initially exported from 3-Matic and Fusion software in stereolithography (STL) format. All MD plates underwent further corrections in Geomagic Wrap software (Artec 3D) to eliminate modelling errors such as holes, intersected triangles, and spikes, which could interfere with subsequent analyses. Next, to ensure comparability across plate types, adaptive remeshing was performed on both MD and GD plates to achieve similar numbers of triangles and vertices. Last, Each plate was exported as STL and Initial Graphics Exchange Specification (IGES) file format for following analysis.

#### Plate-bone shape fit evaluation

Due to inherent irregularities in plate and bone geometry, a signed distance field (SDF) algorithm was employed to quantify shape fit. Specifically, the SDF algorithm measures distances between 2 mesh objects by computing the Euclidean distance from each vertex of the plate geometry to the nearest surface on the bone geometry. First, the MD and GD fixation plates (STL format) and surgically planned bone segments were imported into CloudCompare software. The holes or defects on the bone segments (eg, due to thin bone walls) were repaired prior to shape fit calculation. All vertices of the plate geometry were selected without any down-sampling process, ensuring a high-resolution evaluation of the fit. The SDF calculation was run in the “Compute cloud/mesh distance” module. The underlying bone segments were set as “Reference shape,” while the plates were set as “Compared shape.” The resulting SDF data were shown as histograms, with the number of classes set to 400 for detailed analysis.

Shape fit was reported as 3 metrics: (1) *mean plate-bone distances (Mean_Dist.)*: the mean distance between plate vertices and the bone surface; (2) *vertices <0.5 mm*: the percentage of vertices located within 0.5 mm from the bone surface; and (3) *vertices >1.5 mm*: the percentage of vertices located more than 1.5 mm away from the bone surface. These thresholds (<0.5 mm and >1.5 mm) were established based on clinical considerations and pilot measurements to fully characterise shape fit. A completely close fit is practically impossible over complex bone geometry, yielding pilot mean distances of 0.8 to 1.2 mm for designed plates. Given a standard 1.0-mm plate thickness, the plate’s inner and outer surface vertices naturally distribute around 0.5 mm and 1.5 mm from the bone, respectively. Vertices <0.5 mm and vertices >1.5 mm can capture the extremes of this distribution. Specifically, vertices <0.5 mm indicate the conformity of the plate’s inner surface, while vertices >1.5 mm highlight regions of poor conformity or excess material.

#### Biomechanical performance evaluation

The finite element analysis (FEA) was performed on all MD and GD plates using Ansys 2024 R1 to evaluate their mechanical performance under physiologically relevant load conditions. All fixation plates were meshed with quadratic tetrahedron elements, with an initial mesh size of 0.3 mm for Le Fort I plates and 0.2 mm for genioplasty plates. These mesh parameters were determined based on convergence experiment results and to ensure comparability between paired GD and MD plates. All plates were assigned the material properties of pure titanium.

Accurate and reliable boundary conditions are essential for clinically meaningful results. To balance model simplicity and clinical relevance, static loading rather than cyclic occlusal loading was used for Le Fort I plates. A total occlusal force of 800 N was chosen as a practical compromise between typical chewing forces (∼400 N) and maximal bilateral molar clenching forces (∼1000 N).^10^ Musculature and lateral occlusal force were omitted due to their relatively minor effect. Fixed support was applied to rings attached to the fixed bone segments (upper 8 rings), while upward occlusal force was applied to the rings attached to the movable bone segments (lower 8 rings). For genioplasty plates, a backward traction force of 80 N was estimated based on the established principle that a muscle cross-sectional area of approximately 1 cm² generates approximately 40 N of force.^10^^,^^11^ Similarly, fixed support was applied to rings on the mandibular segments, and the traction force was applied to rings on the chin segments. Additionally, convergence analyses of von Mises stress were performed with a 10% allowable change to ensure accurate and reliable simulation results.

### Statistical analysis

The Shapiro-Wilk test was used to test the normality of intergroup differences of paired variables (shape fit and plate size metrics). For variables that followed a normal distribution, paired *t* tests were used. For variables that did not follow a normal distribution, Wilcoxon signed-rank tests were used. Two-way analysis of variance (plate type × design method) tested interaction effects for shape fit indexes. The effect size was calculated using Cohen’s *d* to assess observed differences. A 2-sided *P* value of .05 was considered. SPSS Statistics (version 29.0; IBM) was used for performing the statistical analyses. Statistical analysis was not performed for maximum von Mises stress and deformation values, as these metrics represent the single highest value observed per plate rather than a distribution across the plate. Therefore, the maximum von Mises stress and deformation were described as ranges and compared with material safety limits.

## Results

### Plate-bone shape fit

No interference was detected between any of the MD/GD plates and the underlying bone. Table 1 summarises the shape fit measurement results. The 2-way analysis of variance test found no interaction effect between plate types and plate design methods (Supplementary Figure S1), so statistical inferences were performed based on the combined dataset (N = 12 pairs).

**Table 1:** Shape fit comparison between MD and GD plates.

Metrics	Plate type	MD		GD		Cohen’s *d*	*P* value
		Mean	SD	Mean	SD		
Mean_Dist. (mm)	Total	1.12	0.28	0.95	0.17	0.75	.009
	LF I	1.23	0.18	1.11	0.05	0.77	
	G	1.01	0.32	0.80	0.09	0.75	
Vertices <0.5 mm (%)	Total	21.0	13.3	27.7	9.0	–0.68	.039
	LF I	12.7	6.1	19.9	4.2	–1.23	
	G	29.4	13.7	35.5	3.6	–0.46	
Vertices >1.5 mm (%)	Total	28.1	17.6	18.6	13.6	0.62	.028
	LF I	35.6	11.7	30.7	6.1	0.47	
	G	20.6	20.2	6.6	4.7	0.83	

Abbreviations: G, genioplasty; GD, generatively designed; LF I, Le Fort I; MD, manually designed.

“Mean_Dist.” shows the mean plate-bone distance. “Vertices <0.5 mm” and “Vertices >1.5 mm” are the percentages of vertices located less than 0.5 mm or more than 1.5 mm away from the underlying bone surface, respectively.

The mean plate-bone distance was 1.12 ± 0.28 mm for MD plates and 0.95 ± 0.17 mm for GD plates (*P* < .05). The GD plates demonstrated a higher percentage of vertices <0.5 mm than MD plates (27.7% vs 21.0%, *P* < .05), suggesting GD plates conform better and closer to the underlying bone. Conversely, the GD plates showed a significantly lower percentage of vertices >1.5 mm (GD, 18.6%; MD, 28.1%; *P* < .05), indicating fewer areas of poor fit existed in GD plates. The absolute values of Cohen’s *d* for all indices ranged from 0.46 to 1.23, showing medium to large effect sizes. Supplementary Figure S2 provides a scalar field comparison of the distribution of vertices <0.5 mm for MD and GD plates. The increased proportion of vertices <0.5 mm in GD plates was primarily located on the connecting bars bridging the osteotomy gap, as well as on the lower transverse connecting bars of Le Fort I plates.

### Biomechanical performance

**Fig. 2:**
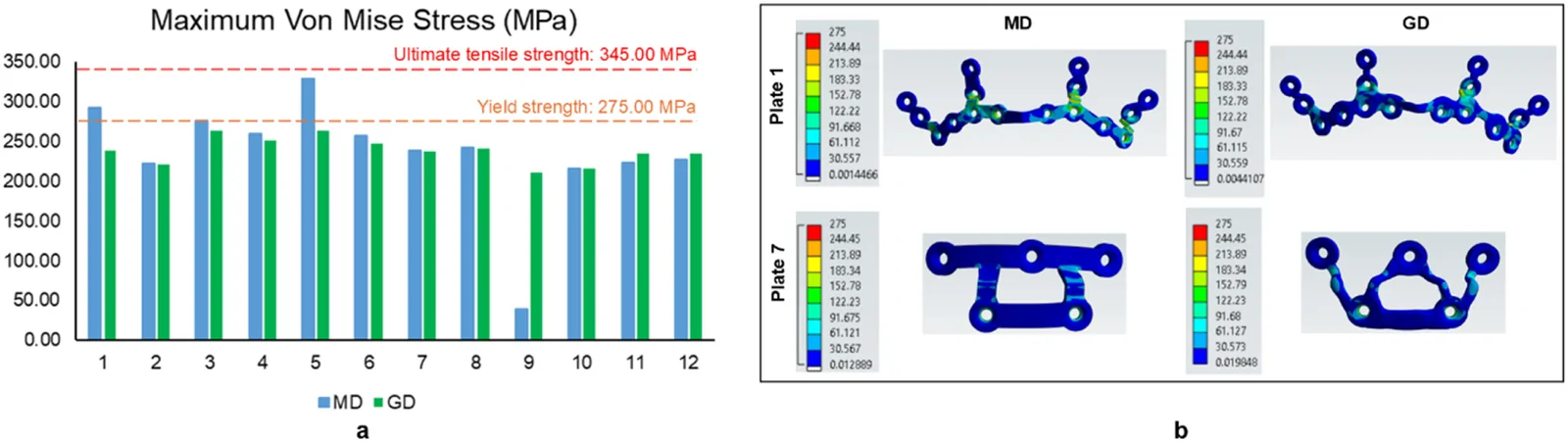
The von Mises stress distribution of fixation plates. a, The maximum von Mises stresses of each plate. Orange and red dotted lines indicate the yield strength and ultimate tensile strength of pure titanium grade 2. b, Le Fort I plate (plate 1) and genioplasty plate (plate 7). The maximum values of scalar bars were unified as 275 MPa. The remaining 10 plates are displayed in Supplementary Figures S3 and S4.

The maximum von Mises stresses of Le Fort I plates ranged from 222.26 to 329.04 MPa in the MD group and 221.63 to 264.21 MPa in the GD group. For the genioplasty plates, the ranges were 39.16 to 239.36 MPa (MD group) and 210.60 to 243.81 MPa (GD group). Except for 3 Le Fort I MD plates (plates 1, 3, and 5), the stresses in all plates remained below the yield strength of pure titanium grade 2 (275 MPa), indicating sufficient mechanical strength to prevent permanent deformation (Figure 2a). The maximum von Mises stresses of the MD plates 1, 3, and 5 remained within the ultimate tensile strength (345 MPa), suggesting the possibility of localised permanent deformation without a risk of fracture. The chromatic view revealed that the distributions of von Mises stress were similar between MD and GD groups (Figure 2b; Supplementary Figures S3 and S4). In both groups, high stress was concentrated along the connectors bridging fixed and movable bone segments, which are the primary load paths of fixation plates. The bending regions of the connectors, whether concave or convex, exhibited significantly higher stress.

**Fig. 3:**
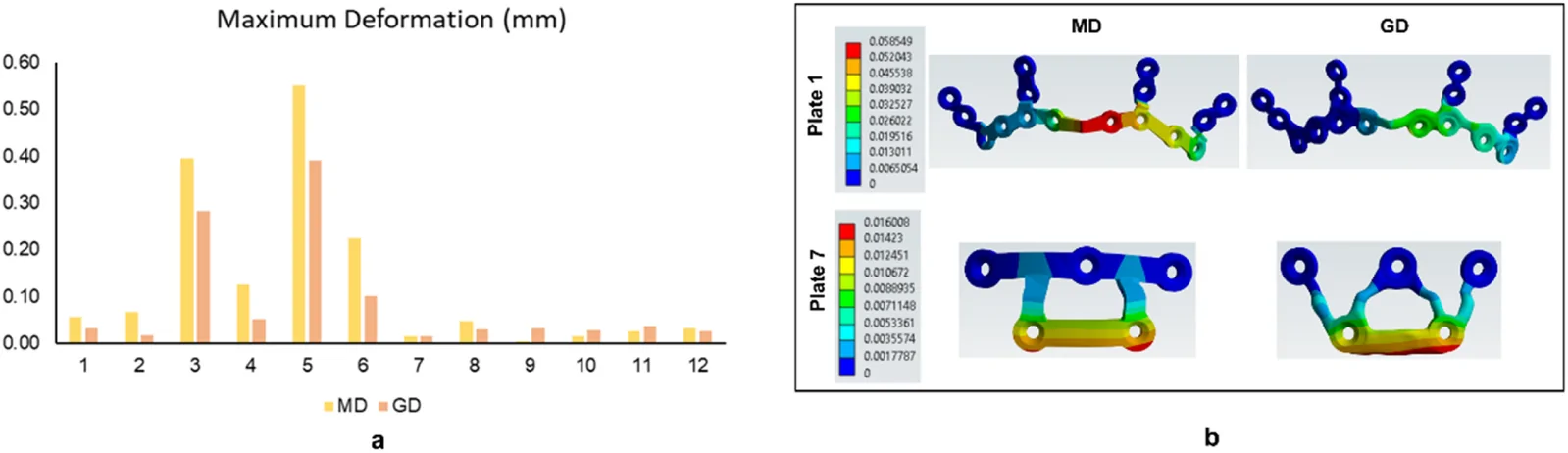
The deformation distribution of fixation plates. a, The maximum deformations of each plate. b, Le Fort I plate (plate 1) and genioplasty plate (plate 7). Scalar bars were adjusted to the same display range for paired plates. The remaining 10 plates are displayed in Supplementary Figure S5.

For genioplasty plates, the maximum deformations were less than 0.05 mm in both MD and GD groups. In contrast, the Le Fort I plates exhibited slightly higher and more variable maximum deformations, ranging from 0.06 to 0.55 mm in the MD group and 0.02 to 0.39 mm in the GD group (Figure 3a). The maximum deformation occurred in similar regions for paired MD and GD plates, with only slight differences in magnitude (Figure 3b; Supplementary Figure S5).

### Plate size

Table 2 compares the surface area and volume of GD plates with their MD counterparts. The mean surface area was significantly smaller in the GD group (1121.0 mm²) compared to the MD group (1385.9 mm²), representing a reduction of approximately 16% (*P* < .001). The mean volumes were 426.8 mm³ in the GD group and 510.0 mm³ in the MD group, reflecting a significant reduction of 18% (*P* < .05). The genioplasty GD plates exhibited a notably greater reduction in surface area and volume than the Le Fort I GD plates.

**Table 2:** Basic properties of MD and GD plates.

Variable	Plate type	MD		GD		Absolute difference	Relative difference, %	*P* value
		Mean	SD	Mean	SD			
Surface area (mm^2^)	Total	1385.9	482.1	1121.0	473.3	217.5	16	<.001
	LF I	1692.8	56.9	1502.9	103.7	227.0	13	
	G	747.1	91.8	626.6	122.8	199.5	27	
Volume (mm^3^)	Total	510.0	196.5	426.8	234.0	89.3	18	.013
	LF I	674.8	98.3	645.0	78.1	50.5	7	
	G	293.4	95.9	231.2	60.8	111.5	38	

Abbreviations: G, genioplasty; GD, generatively designed; LF I, Le Fort I; MD, manually designed.

Absolute difference = MD – GD; relative difference = [(MD – GD) / MD] × 100 (%).

### Required design time

The required time for each procedure in the GD workflow was recorded (Table 3). GD computation, which is an automatic process, took 29.5 ± 2.3 minutes and 20.0 ± 3.9 minutes for Le Fort I and genioplasty plates, respectively. The average hands-on time was 94.3 minutes for Le Fort I plates and 45.0 minutes for genioplasty plates. Additionally, designing surgical guides for the Le Fort I osteotomy and genioplasty took an average of 29.0 minutes and 24.3 minutes, respectively. Overall, by adopting the GD workflow, the manual work for designing a set of PSIs (a fixation plate and a surgical guide) cost approximately 125.0 minutes for a Le Fort I osteotomy case and 69.3 minutes for a genioplasty case. In contrast, conventional MD workflow typically requires 180 to 300 minutes for a Le Fort I case and 120 to 180 minutes for a genioplasty case based on self-reported data from oral and maxillofacial surgery trainees at our centre.

**Table 3:** Required design time for plate and surgical guide in GD workflow (unit: minutes).

Plate type	GD							MD
	Fixation plate					Surgical guide	Total	
	Adjust screws	Edit mesh	GD computation	Postprocess	Hands-on time			
Le Fort I	27.0 ± 6.4	40.7 ± 8.4	29.5 ± 2.3	28.3 ± 6.9	94.3 ± 14.2	29.0 ± 6.2	125.0 ± 15.2	180-300
Genioplasty	13.5 ± 1.9	15.0 ± 3.0	20.0 ± 3.9	16.5 ± 3.8	45.0 ± 7.3	24.3 ± 1.0	69.3 ± 7.7	120-180

Abbreviations: GD, generatively designed; MD, manually designed.

Hands-on time = Adjust screws + Edit mesh + Postprocess, representing hands-on design time for a fixation plate. Total = Hands-on time + Surgical guide, indicating overall hands-on time for designing a set of PSIs. The MD times were based on self-reported estimates from both junior and senior oral and maxillofacial surgery trainees at our centre. The design times for the fixation plate and the surgical guide were both included.

## Discussion

Although passively fitting the underlying bone is a core requirement for fixation plate design, objective quantification of plate-bone shape fit has not been reported.^9^ This study introduced a quantifiable shape fit index as an indirect indicator of bone segment repositioning accuracy in surgery. Our results demonstrated that GD plates outperformed MD plates across all 3 shape fit metrics (Table 1), indicating the GD workflow yields superior plate conformity to bone shape than manual design. This improvement was most evident in anatomically complex regions, such as steep bone steps and concave surfaces. It demonstrated that the generative design workflow could adapt connector curvature to the underlying anatomy more effectively than manual straight-line approaches (Supplementary Figure S2). While manual modification of connector pathways is possible, achieving similar conformity is challenging and time-consuming. Notably, both design methods struggled with highly irregular bone surfaces, as seen in the generally poorer conformity of Le Fort I plates versus genioplasty plates (Table 1). Despite the statistically significant advantage of GD plates in shape fit, the direct impact on surgical accuracy remains to be validated clinically. However, these findings suggest that GD plates may achieve at least equivalent, if not improved, surgical accuracy compared with MD plates. Improved shape fit may be particularly beneficial for controlling pitch movement, as prior studies have shown consistently greater surgical errors in this dimension during maxillary repositioning.^12^, ^13^, ^14^, ^15^ Closer conformity at step areas could help restrict undesired tilting of bone segments. Additionally, the lower variance in shape fit observed with GD plates highlights the robustness and reproducibility of the generative design workflow. The clinical benefits of these improvements should be confirmed in future studies.

We evaluated 2 primary biomechanical metrics: maximum von Mises stress and maximum deformation. Clinically, values of maximum von Mises stress exceeding the yield (275 MPa) or ultimate strength (345 MPa) of titanium indicate high risk of permanent deformation and material failure, respectively. Maximum deformation reflects potential instability at the osteotomy site, which may compromise bone healing or fixation stability.^16^^,^^17^ In our FEA, only 3 Le Fort I MD plates (plates 1, 3, and 5) showed von Mises stresses above the yield strength. For plates 1 and 3, the peak stresses were localised at artificially sharp corners resulting from manual modelling inaccuracies, which are unlikely to persist after 3-dimensional printing (Supplementary Figure S6). Therefore, these stress concentrations are not expected to cause permanent deformation clinically. However, plate 5’s stress was concentrated at a small fillet radius—an area prone to fatigue failure.^17^ In contrast, GD plates, characterised by smoother, round-edged geometries, eliminated such weak points through iterative computational optimisation.

Both GD and MD plates showed similar deformations: less than 0.05 mm for genioplasty plates and no more than 0.55 mm for Le Fort I fixation plates (Figure 3a), both within clinically acceptable limits for maintaining stability during bone healing.^18^ Overall, these results confirm that GD plates offer comparable biomechanical strength to MD plates, despite their smaller size.

Conventional MD plates may have been overengineered. Notably, 1 MD genioplasty plate (plate 9) presented extremely low maximum von Mises stress (39.2 MPa) and deformation (0.005 mm), suggesting the plate was quite rigid. Overengineering increases design and manufacturing complexity, as well as cost (as it requires more titanium powder for fabrication), and may even compromise clinical outcomes. Excessively rigid implants can cause stress shielding, inhibiting natural bone remodelling and healing.^19^ Larger implants are also associated with higher risks of infection, readmission, and reoperation^20^ and may result in patient discomfort or difficulty with plate removal.

Previous research has explored optimisation strategies to address these issues. Liu et al^21^ used topological optimisation to design a “V”-shaped fixation plate for mandibular angle fractures, showing better biomechanical behaviour than conventional 1 or 2 mini-plates. Wang et al^22^ customised an asymmetric fixation plate for bilateral sagittal split osteotomy of a hemifacial microsomia case to improve resistance to relapse compared with conventional plates. Although clinical outcomes of these optimised designs remain to be fully reported, incorporating biomechanical considerations into PSI design clearly holds promise for improving surgical predictability and stability, particularly in complex OGS cases.

Compared with manual design, the GD approach systematically incorporates biomechanical constraints during the initial design stage. GD plates achieved similar or superior mechanical performance while minimising unnecessary bulk and material usage. This study demonstrated that the GD workflow effectively addresses the overengineering issue.^6^

Manual design times were not systematically recorded due to the retrospective nature of the cases, but historical data provide context. At our centre, surgeons proficient in PSI design using 3-matic required 3 to 5 hours for Le Fort I and around 2 to 3 hours for genioplasty plates. Another study reported mean design times of 102.5 minutes (Le Fort I) and 35 minutes (genioplasty) by medical engineers, excluding data preprocessing and screw positioning steps.^23^ In comparison, the GD workflow averaged 94.3 minutes for Le Fort I and 45.0 minutes for genioplasty plates, with all steps after VSP included. During the process, screw positioning accounted for 27.0 and 13.5 minutes, respectively (Table 3). Thus, the GD workflow reduced design time by roughly one-third to one-half. Prospective studies are warranted to confirm these findings.

This study has several limitations. The study included a modest sample size (N = 12) without a priori power calculation. Effect sizes were reported to guide future trials to verify the superiority of GD plates. The FEA results in this study should be cautiously interpreted as modelling and boundary conditions were selectively simplified to balance result validity and high computational costs (eg, lack of screw-bone contact, lack of cyclic loading simulation for fatigue analysis). This study is only a preclinical, in silico comparative study without validation on postoperative outcomes, so the clinical impact of the investigated features on accuracy, healing, and complications remains unclear. Prospective clinical studies are planned, evaluating primary outcomes such as 3-dimensional positional and rotational accuracy versus VSP, early relapse, and complication rates, as well as secondary endpoints like PSI design time, operative time, and cost-effectiveness.

## Conclusion

GD fixation plates demonstrated superior shape fit, smaller size, and significantly reduced design time compared with MD plates while maintaining equivalent mechanical performance. These advantages suggest that GD plates may improve clinical outcomes, patient comfort, and workflow efficiency. Prospective clinical trials are warranted to validate these findings.

## Author contributions

*Conceptualisation:* Cheng, Nayak, Leung. *Methodology:* Cheng, Nayak. *Project administration:* Leung. *Data curation:* Cheng, Nayak. *Analysis and interpretation of the data:* Cheng, Nayak, Leung. *Writing—original draft:* Cheng, Nayak. *Writing—review and editing:* Cheng, Nayak, Leung. *Supervision:* Leung. All authors have agreed to publish this final version of the manuscript.

## Ethics statement

The study was approved by the Institutional Review Board of the University of Hong Kong/Hospital Authority Hong Kong West Cluster (HKU/HA HKW IRB UW 24-764).

## Funding

The work is supported by the General Research Fund of Hong Kong (17112525).

## Conflict of interest

None disclosed.

## Appendix Supplementary materials

Image, application 1

## Footnotes

Supplementary material associated with this article can be found in the online version at doi:10.1016/j.identj.2026.111095.
