# Supplementary material for: Feasibility of Generatively Designed Patient-Specific Implants for Orthognathic Surgery: An In Silico Comparative Study
Source: Int Dent J. 2026 Sep 2;76(5):111095. doi: 10.1016/j.identj.2026.111095 (PMC13570290; doi:10.1016/j.identj.2026.111095)
Supplement: Supplementary file 1 [file mmc1.docx]

Supplementary materials of the manuscript titled **‘Feasibility of Generative Designed Patient-Specific Implants for Orthognathic Surgery: An In Silico Comparative Study**’

**Supplementary Fig 1. Profile plots of two-way ANOVA tests.** **a.** Mean plate-bone distance. **b.** Vertices<0.5mm. **c.** Vertices >1.5mm.

**Supplementary Fig 2. Scalar field display of ‘vertices< 0.5 mm’ of fixation plates (Back view).** **a.** The Le Fort I MD and GD plates. **b.** The genioplasty MD and GD plates. All vertices located within 0.5mm away from the underlying bone were highlighted. Specific proportion values were annotated on each figure.

**Supplementary Fig 3. The Von Mises stress distribution of Le Fort I fixation plates.** MD: manual design; GD: Generative design.

**Supplementary Fig 4. The Von Mises stress distribution of genioplasty fixation plates.** MD: manual design; GD: Generative design.

**Supplementary Fig 5. The deformation distribution of fixation plates.** MD: manual design; GD: Generative design; Scalar bars were adjusted as same display range for paired plates.

**Supplementary Fig 6. The locations of the maximum Von Mises stresses and geometry weaknesses for Le Fort I MD fixation plates (Plates 1, 3 and 5).** Plate 1 showed a folded sharp corner. Plate 3 showed a narrow sharp groove. Plate 5 showed a concave junction area.
